# Electrospun Poly (Vinylidene Fluoride-Co-Hexafluoropropylene) Nanofiber Membranes for Brine Treatment via Membrane Distillation

**DOI:** 10.3390/polym15122706

**Published:** 2023-06-16

**Authors:** Amjad Albiladi, Lassaad Gzara, Hussam Organji, Nazeeha S. Alkayal, Alberto Figoli

**Affiliations:** 1 Chemistry Department, Faculty of Science, King Abdulaziz University, Jeddah 21589, Saudi Arabia; amjadhazza@gmail.com (A.A.); nalkayal@kau.edu.sa (N.S.A.); 2 Center of Excellence in Desalination Technology, King Abdulaziz University, Jeddah 21589, Saudi Arabia; haorganji@kau.edu.sa; 3 Institute on Membrane Technology (ITM-CNR), Via P. Bucci 17c, 87036 Rende, CS, Italy; a.figoli@itm.cnr.it

**Keywords:** polyvinylidene fluoride-co-hexafluoropropylene (PVDF-HFP), electrospinning, nanofibers, hydrophobic membrane, membrane distillation

## Abstract

The major challenge for membrane distillation (MD) is the membrane wetting resistance induced by pollutants in the feed solution. The proposed solution for this issue was to fabricate membranes with hydrophobic properties. Hydrophobic electrospun poly (vinylidene fluoride-co-hexafluoropropylene) (PVDF-HFP) nanofiber membranes were produced for brine treatment using the direct-contact membrane distillation (DCMD) technique. These nanofiber membranes were prepared from three different polymeric solution compositions to study the effect of solvent composition on the electrospinning process. Furthermore, the effect of the polymer concentration was investigated by preparing polymeric solutions with three different polymer percentages: 6, 8, and 10%. All of the nanofiber membranes obtained from electrospinning were post-treated at varying temperatures. The effects of thickness, porosity, pore size, and liquid entry pressure (LEP) were studied. The hydrophobicity was determined using contact angle measurements, which were investigated using optical contact angle goniometry. The crystallinity and thermal properties were studied using DSC and XRD, while the functional groups were studied using FTIR. The morphological study was performed with AMF and described the roughness of nanofiber membranes. Finally, all of the nanofiber membranes had enough of a hydrophobic nature to be used in DCMD. A PVDF membrane filter disc and all nanofiber membranes were applied in DCMD to treat brine water. The resulting water flux and permeate water quality were compared, and it was discovered that all of the produced nanofiber membranes showed good behavior with varying water flux, but the salt rejection was greater than 90%. A membrane prepared from DMF/acetone 5-5 with 10% PVDF-HFP provided the perfect performance, with an average water flux of 44 kg.m^−2^.h^−1^ and salt rejection of 99.8%.

## 1. Introduction

**Figure 1:**
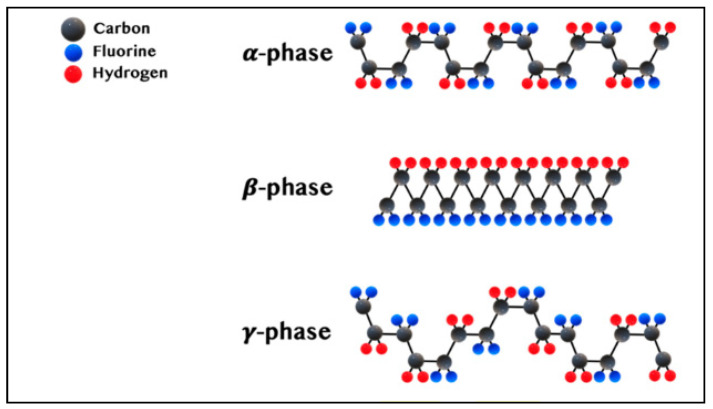
The essential phases of PVDF [22].

Through the ages, water scarcity has remained the biggest problem in the world that is faced by many countries and is still one of the most important global challenges to date. Despite 70% of the Earth’s surface being water, only less than 3% of that is pure water, and it is unequally distributed around the world. However, natural underground water is threatened by depletion due to increased demand without control [1,2,3]. To address this rapidly growing issue, a group of scientists have begun to look into producing freshwater using various low-cost and environmentally friendly technologies [4,5]. Researchers have focused on seawater desalination and wastewater treatment to alleviate the demand for freshwater, and these two sources are largely abundant. Water desalination operations have consumed a lot of energy for a long time, indicating that they are very costly processes [6,7]. Membrane separation technologies have been extensively researched in recent years due to their unique characteristics such as the high quality of its treated water and low operational and energy costs [8]. Membrane-based technologies are classified into microfiltration (MF), ultrafiltration (UF), nanofiltration (NF), reverse osmosis (RO), forward osmosis (FO), and membrane distillation (MD), depending on the diameter of pores and the membrane separation properties. Furthermore, the main difference between all previous membrane-based techniques and MD is that MD is a thermally driven process, while the others are pressure-driven operations [8]. Among all these membrane-based techniques, the membrane distillation (MD) technique has received great attention due to its various advantages compared to other techniques, including: (i) high salt rejection (>99.9%), (ii) significantly larger pore size, (iii) lower fouling sensitivity, (iv) reduced feed salinity sensitivity, (v) ability to use low-grade heat (40–80 °C), (vi) the fact that renewable energy can be used to heat the feed solution, which can be generated by waste heat from cogeneration plants and solar energy [9,10,11]. The membrane distillation (MD) process is based on the difference in vapor pressure between feed and permeation solutions created by the temperature gradient [12]. A large number of studies have preferred the use of polymeric membranes due to their low cost, high separation selectivity, and good separation performance. Typical examples of polymers utilized in MD research are polyvinylidene fluoride (PVDF), polyethersulfone (PES), polyethylene (PE), polypropylene (PP), polyvinyl alcohol (PVA), polystyrene (PS), and polytetrafluoroethylene (PTFE). PVDF is a semicrystalline fluorocarbon homopolymer that possesses special properties such as excellent chemical resistance, hydrophobicity, and thermal and mechanical properties. In addition, due to PVDF having good solubility in some common polar organic solvents, it is easy to handle [1,13,14]. All these properties make PVDF an excellent candidate for membrane fabrication that is utilized for different purposes such as water purification [15,16], lithium-ion batteries [17,18,19], gas separation [20], etc. PVDF polymer is categorized as a piezoelectric material. The crystalline polymorphs of PVDF are formed depending on the various processing methods. There are five crystalline polymorphs of PVDF, which are α-, β-, γ-, δ-, and ε-phases. The common three phases are the main phases of PVDF that are explained in Figure 1. Those five crystal structures are divided into polar and nonpolar phases. Polar phases include β- and γ-phases, which determine PVDF’s ferroelectric and piezoelectric properties. Among these structures, the β-phase has a higher polarity than the others (γ and δ phases), but they also have polar unit cells. The piezoelectricity property of the β-phase is better than that of the γ-phase because of the presence of a gauche bond, which appears in every fourth repeat unit [21,22,23,24].

Poly (vinylidene fluoride-co-hexafluoropropylene) (PVDF-HFP) is a copolymer resulting from vinylidene fluoride (VDF) and hexafluoropropylene (HFP) when they undergo emulsion polymerization. The incorporation of the HFP group in VDF polymer is responsible for improving its properties because the fluorine segment has a high hydrophobicity and lower polarity. Thus, PVDF-HFP possesses higher solubility (especially in organic solvents), higher hydrophobicity, better mechanical strength, a lower glass transition temperature, lower crystallinity, and higher free volume compared with PVDF [25,26].

PVDF-HFP nanofiber membranes have been produced using several techniques for different applications, but among these methods, electrospinning has proven to be effective in producing membranes with high efficiency and better properties.. Briefly, it produces the preferred phase of PVDF (β-phase), which enhances the piezoelectricity properties of films, due to polymer jet elongation, and gives a great amount of voltage when mechanical stress, strain, and bending activities are applied. Hence, high-performance polymer films are usually produced by electrospinning, which involves a high electric field applied to the polymer [22].

Electrospinning is a promising simple method for fabricating nanofiber membranes for an extended variety of applications. It has three main parts: (i) a high-voltage supply, (ii) a spinneret with the polymer solution, (iii) and a grounded collector. When a high voltage is applied, the fine fibers will be produced from the polymer solution, where the polymer jet comes through the needle of the syringe and spins until being collected in the collector as fibers of a few nanometers to micrometers [8,27].

The desired membrane structure can be controlled by tuning its preparation conditions, including the chemical structure of polymeric solutions and operational and environmental parameters. The morphology of the membrane depends on solvents since they influence the kinetic and thermodynamic parameters of solvent–polymer interaction. 

The efficiency of desalination techniques that operate with membranes depends on the membrane’s properties. Salt rejection and water flux are the basic properties that must be considered during the desalination process. Flux and salt rejection are affected by porosity, pore size, and the high specific surface area of the electrospun nanofiber membrane. The best MD membranes usually have high liquid entry pressure (LEP), low thermal conductivity, low fouling rate, excellent chemical and thermal stability, and excellent mechanical strength. In the last few years, membranes have been improved until membrane-based desalination techniques have become operational with minimum energy [1,28,29]. There are different types of MD operation, namely, direct-contact membrane distillation (DCMD), vacuum membrane distillation (VDM), air gap membrane distillation (AGMD), and sweeping gas membrane distillation (SGMD).

Direct-contact membrane distillation (DCMD) is the simplest form of MD, where both sides of the feed and permeate are in direct contact through the process. This procedure is driven by a difference in temperature between the feed side and permeate side, which leads to a difference in vapor pressure between them, which drives the flux across the membrane [1,9]. The salt rejection factor of the PVDF-HFP copolymer nanofiber membrane was found to be higher than that of the PVDF homopolymer nanofiber membrane when they were employed in DCMD [30]. The electrospun PVDF nanofiber membrane was tested to treat a 3.5 wt% NaCl solution with DCMD, and the water permeation flux was about 21 kg m^−2^ h^−1^ [31]. A novel PVDF beaded-nanofiber membrane was used in DCMD and exhibited that the insertion of beads inside nanofibers led to an increase in the hydrophobicity property and water permeability of membranes by 34% with constant water flux compared to the performance of commercial PVDF [32]. Further research suggested that the presence of silica nanoparticles in polymer structures is capable of enhancing water flux during DCMD experiments, even though it is responsible for reducing the hydrophobicity property of membranes. The addition of silica nanoparticles enhanced the water flux of PVDF-HFP nanofiber membranes by 53% compared to PVDF-HFP nanofiber membranes without silica nanoparticles [33].

This work aims to fabricate PVDF-HFP nanofiber membranes using the electrospinning technique for membrane distillation applications. In this work, a dimethylformamide (DMF)/ acetone mixture was chosen as a solvent to prepare the polymeric solutions. The effect of the DMF/acetone mixture composition and the polymer solution concentration on the morphology, crystallinity, and hydrophobicity of the developed electrospun PVDF-HFP nanofibers was assessed. Moreover, the performance, in terms of flux and salt rejection, of the nanofiber membranes was assessed using the direct-contact membrane distillation system (DCMD).

## 2. Experimental Section

### 2.1. Materials

Poly (vinylidene fluoride-co-hexafluoropropylene) PVDF-HFP Solef^®^21216 (Solvay Specialty Polymers, Bollate, Italy; Mw: 600,000 g.mol^−1^) was dried for a few minutes at nearly 60 °C before use to remove any moisture. Acetone and N, N-Dimethylformamide (DMF) were used as solvents in different composition ratios, whereas lithium chloride (LiCl, anhydrous, 99%) was used as an additive. Except for PVDF-HFP, all chemicals were purchased from Sigma-Aldrich Company and used without further purification,

### 2.2. Dope Solutions Preparation

PVDF-HFP dope solutions were prepared for electrospinning by dissolving a specific percentage of PVDF-HFP polymer in 50 g of DMF and acetone solutions prepared with various weight ratios (6:4, 5:5, and 4:6 wt%). A suitable amount of LiCl (0.1%) was added to all dope solutions to develop the dope electrospin ability. Table 1 shows the preparation parameters of all dope solutions which were stirred for periods varying from an hour to one day at 60 °C until the polymer fully dissolved. The homogenous dope solutions were degassed at room temperature overnight before electrospinning.

### 2.3. Membrane Fabrication

All dope solutions underwent electrospinning to produce nanofiber membranes. Moreover, the efficiency of methods used to fabricate a hydrophobic membrane with desired properties, such as high porosity, narrow pores, and long-term performance, was tested by fabricating membranes from one dope solution (FA 4-6 P10) using two methods, namely, electrospinning and the nonsolvent-induced phase separation (NIPS) method.

#### 2.3.1. Electrospinning PVDF-HFP Dope Solutions

All membranes were produced using a high-voltage power supply (22 kV) with a fixed needle tip (0.7 mm), and the distance between the needle and collector was 17 cm. Furthermore, the dope solution volume was 10 mL, and the flow rate was 1 mL/h for all polymeric solutions, except for the FA 6-4 P8 and FA 6-4 P10 polymeric solutions, whose flow rates were reduced to 0.5 and 0.8 mL/h, respectively. Heat treatment was applied to all electrospun membranes.

Post-heat treatment for all electrospun membranes was applied to remove the residual solvent by putting them in an oven at 50 °C for an hour. Then, the nanofiber membranes were put between two glass plates and left in the oven at 100 °C for a day. After that, further heat treatment was applied, which started at 140 °C for an hour under pressure; then, the pressure was removed, and the temperature was reduced by 10 °C per hour until it reached 100 °C. Then, the membranes were left in the oven for a day. Pure PVDF-HFP was not subjected to post-heat treatment before characterization.

#### 2.3.2. The Nonsolvent-Induced Phase Separation (NIPS) Method

Commercially successful membrane separation can be attributed to the development of asymmetric membranes. The phase inversion process can make asymmetric membranes. Phase inversion is phase separation followed by solidification. In nonsolvent-induced phase separation (NIPS), a cast polymer solution is immersed in a nonsolvent bath. Nonsolvent enters the polymeric matrix, displacing solvent; the cast film is deepened in nonsolvent, inducing phase separation.

To make a comparison between electrospinning and NIPS methods that are used to fabricate hydrophobic membranes, the dope solution FA 4-6 P10 was used for electrospinning, as previously mentioned, whereas during the NIPS method, the same dope solution underwent a casting process at room temperature using a glass plate and a casting blade. Then, it was kept for an hour before the coagulation bath to allow the solvents to evaporate. A coagulation water bath was applied to enable solvent and nonsolvent exchange in the membrane matrix. The NIPS membrane was not subjected to post-heat treatment.

### 2.4. Characterizations of PVDF-HFP Nanofiber membranes

The chemical and physical properties of electrospun nanofiber membranes were characterized using different techniques and mathematical relations.

#### 2.4.1. Thickness, Mean Pore Size, and LEP

The membrane thickness was measured in five different locations for all membranes using a digital micrometer and the average of those values was taken. The mean pore size (*r_m_*) was calculated according to Guerout–Elford–Ferry’s equation [15]:

$$r_{m}=\sqrt{\frac{\left(2.9-1.75\varepsilon \right)\left(8\eta lQ\right)}{\varepsilon A\;∆P}}$$

where *η* is the viscosity of water which is equal to 8.9 × 10^−4^ Pa s; ε and *l* are the porosity (%) and the thickness (µm) of the membrane, respectively; Δ*P* is the operation pressure (Pa); *Q* is the volume of permeate water per unit of time (m^3^s^−1^); and *A* is the effective area of the membrane (m^2^) [15].

Liquid entry pressure (LEP) was measured for all membranes using a cylindrical pressure filtration cell with an effective surface area of 25.12 cm^2^. The PVDF-HFP membrane was put on the bottom surface of the filtration cell; then, it was pressed with 100 mL of distilled water and air, which were used as feed. The pressure began at 0.1 bar and was increased every 5 min or 30 s, depending on the water flux, until it reached 2.5 bar. Then, the pressure was decreased by 1 bar every 5 min or 30 s until it reached the starting pressure. In both forward and backward directions, before increasing the pressure, the mass of passed water was taken manually. LEP is defined as the pressure at which the first drop of water passes through the membrane and appears on the permeate side [34].

#### 2.4.2. Porosity and Water Uptake

Firstly, the porosities and water uptake of PVDF-HFP nanofiber membranes were specified by the immersion of 1 cm^2^ of each membrane in pure water and kerosene separately for 24 h at room temperature. The wet weight was measured after wiping the excess amount of water or kerosene from the membranes’ surface, whereas the dried weight was obtained after drying the membrane pieces in an oven at 50 °C overnight. Then, the porosity (P%) and water uptake (WU%) were calculated based on the following equations:

$$P\%=\frac{\frac{W_{w}-W_{d}}{\rho_{s}}}{\frac{W_{w}-W_{d}}{\rho_{s}}+W_{d}/\rho_{p}}\times 100$$

$$\mathrm{WU}\%=\frac{W_{w}-W_{d}}{W_{d}}\times 100$$

where W_w_ and W_d_ are the wet and dried weights of the membrane (g), respectively; ρ_s_ is the density of solvent (kerosene) used to stock membranes (g cm^−3^); and ρ_p_ is the density of polymer (PVDF). In porosity calculations, the wet and dried weights of membranes were taken with kerosene, but in water uptake measurements, the wet and dry weights of membranes were taken with distilled water [15,35].

#### 2.4.3. Contact Angle (CA)

For the evaluation of the membrane surface hydrophobicity, the water contact angle was measured for each membrane with an optical tensiometer instrument (Theta). In all measurements, distilled water was used. To reduce the experimental mistakes, the contact angles were measured for all membranes in three locations and the average number was reported.

#### 2.4.4. DSC, FTIR, and XRD

Differential scanning calorimetry (DSC, 131 Evo) was used to determine the thermal properties and the crystal structure variation of all PVDF-HFP electrospun nanofibers membranes at a heating and cooling rate of 5 K/min within the temperature range of 298–523 K.

The surface chemical compositions and functional groups of all membranes were analyzed using Cary 360 Fourier-transform infrared spectroscopy (FTIR) and all FTIR spectra were recorded using the attenuated total reflection (ATR) technique in the wavelength range of 4000–400 cm^−1^.

X-ray diffraction (XRD) patterns were studied using a Bruker D8 Advance with Cu Kα radiation (wavelength 1.5418 Å) at 40 kV and 40 mA. The patterns were collected between 2θ of 10° and 80°, and the scan speed was 1.5 degree/min.

#### 2.4.5. Morphological Studies: AFM

Atomic force microscopy (AFM) was used to study the morphological structure of the PVDF-HFP electrospun nanofiber membranes. The roughness and surface morphology of the PVDF-HFP membranes were analyzed using AFM. A small piece of each membrane was put on a glass substrate to be inserted into the AFM device. The AFM took an image of the membrane surface at a size of 10 µm × 10 µm using the Gwyddion program which exhibits the roughness as peaks and valleys above the surface. The surface roughness can be studied in different terms such as mean roughness (R_a_), the root mean square of the Z data (R_q_), and the mean difference between the five highest peaks and lowest valleys (R_z_). However, the average roughness is the commonly utilized parameter for evaluating the surface roughness, where its values are usually obtained by the Nanosurf CoreAFM software (https://www.nanosurf.com/en/software/coreafm, accessed on 8 June 2023) [36,37,38].

#### 2.4.6. Membrane Performance: DCMD Membrane Tests

**Figure 2:**
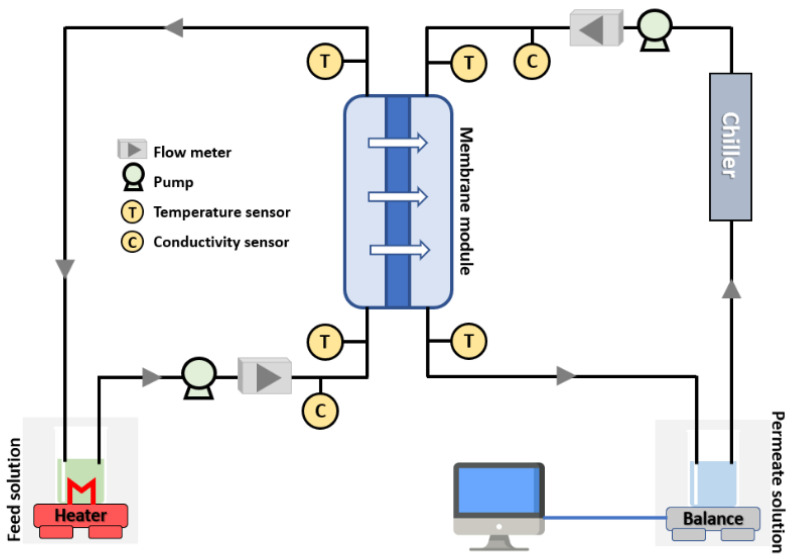
Schematic diagram of the DCMD system.

DCMD was used for evaluating the electrospun PVDF-HFP membrane performance during the membrane distillation process. The DCMD setup is shown in Figure 2, where it contains two pumps, a feed/permeate tank, a digital balance, a flow meter, a conductivity meter, a heater, and a condenser. The feed tank was filled with real brine solution brought from a local seawater reverse osmosis (SWRO) plant. The feed side temperature was 70 ± 3 °C and the conductivity was 72 mS/cm. The permeate tank was filled with 1.5 L of distilled water with a conductivity below 2 µS/cm and then placed over the balance. The temperature of the permeate side was kept constant at 20 ± 3 °C. The experiments were conducted at a constant pressure of 0.45 bar on both the permeate and feed sides and the flow rate was 3 L/min. The steam of water passed from the hot feed side through the pores of the membrane to the permeate side.

The mass of transported water was recorded manually every 5 min for 2 h. Then, the flux of water (kg m^−2^ h^−1^) was calculated as:

$$J=\frac{∆m}{A\;∆t}$$

where A is the effective area of the membrane (equal to 0.0478 m^2^) and t is the time in hours [39]. In addition, the conductivity was constantly measured to evaluate the salt rejection which was calculated by the following equation:

$$R=\left(1-\frac{C_{p}\left(\frac{v_{0}}{v_{p}}+1\right)}{C_{f}}\right)\times 100$$

where *C_p_* and *C_f_* represent the solute concentration in both permeate and feed solutions (mol L^−1^), respectively; *v*_0_ and *v_p_* represent the initial water volume and final water volume of the permeate at a specific time [40].

## 3. Result and Discussion

### 3.1. Dope Solution Characterizations

Hansen solubility parameters (HSP) are utilized to estimate the possibility of miscibility of two or more materials. Thus, the HSP can be written as:

$$R_{A\text{-}B}=\sqrt{{\left(\delta_{dA}-\delta_{dB}\right)}^{2}+{\left(\delta_{pA}-\delta_{pB}\right)}^{2}+{\left(\delta_{hA}-\delta_{hB}\right)}^{2}}\;$$

where *δ_d_*, *δ_p_*, and *δ_h_* represent the dispersion force between molecules, polar cohesion, and hydrogen bond force parameters, respectively; these are listed in Table 2 for the solvents and the polymer used in this study. *A* is a symbol of the polymer (PVDF-HFP) and *B* is a symbol of the solvent or nonsolvent. *R_A-B_* represents the distance from the solvent location to the center of the solute molecule. Thus, it can be compared with the solubility radius of a solute (*R*_0_), such as a polymer in this case, which creates a new term named the relative energy density (*RED*).

The relative energy density (*RED*) gives the radius of interaction that describes the relative compatibility between two components, and it can be written as [26]:

$$RED=\frac{R_{A\text{-}B}}{R_{0}}$$

There are three cases of *RED* results:

i. If *RED* < 1, it means the polymer will not dissolve.
ii. If *RED* > 1, it means the polymer will dissolve.
iii. If *RED* = 1, it means the polymer may swell.

Indeed, all binary solvents’ *RED* values were less than one, which confirms the ability of solvents to dissolve the polymer. The production of the membranes in electrospinning depends on several factors, including the rate of solvent evaporation, which is measured using the vapor pressure concept. Vapor pressure always indicates the volatility of the solvent and can be calculated according to Raoult’s law by the following equation:

$$P=P_{a}X_{a}+P_{b}X_{b}$$

The purpose of utilizing binary solvents is to control the vapor pressure, which in turn controls the solvent’s evaporation rate during the electrospinning process. Since acetone has a high vapor pressure (25 kPa), it is usually mixed with the major solvent to accelerate solvent evaporation and fabricate a dry membrane. The high vapor pressure of the binary solvents ensures the possibility of using dope solutions in electrospinning and producing a dry membrane. The vapor pressure of the binary solvents (i.e., DMF/acetone) is listed in Table 3; it increased with the increasing acetone weight fraction.

The viscosity and surface tension of solutions are critical properties in the electrospinning process, so they should be in an appropriate range where the process cannot proceed under or above this range. At high viscosity, polymer extrusion is impossible, while at low viscosity, the droplets of the polymer may interrupt. The surface tension usually determines the voltage used in electrospinning that is required to be overcome. It is determined by solvent ratios and polymer percentage [8]. As shown in Table 4 in the DMF/acetone (FA 6-4 Px, x = 6, 8, 10%) dope solutions, the viscosity increased with increasing polymer content to be 70, 175, and 405 cp, respectively, but it decreased when fixing polymer percentage and increasing the weight fraction of acetone. Overall, in this study, the suitable viscosity for electrospinning was found in a range between 70 and 400 cp for all DMF/acetone dope solutions. Viscosity and surface tension values were found in the appropriate range for electrospinning that was reported in the literature [41,42].

### 3.2. Membrane Surface Characterizations

Concerning the structural properties of the membrane, the thickness, porosity, pore size, and contact angle were investigated to ensure that the membranes were suitable for water treatment applications. Table 5 shows the thickness and pore size results of PVDF-HFP nanofiber membranes. The polymer percentage used in the preparation of the nanofiber membranes affected the thickness of these membranes. For example, the FA 6-4 P6 membrane had a thickness of 64.8 µm, which increased as the polymer percentage increased from 8% to 10%. The FA 6-4 P6 and FA 6-4 P8 membranes had a thickness of 184 and 251 µm, respectively. Furthermore, the difference in thickness of these three membranes was a little small between the FA 6-4 P6 and FA 6-4 P8 membranes, but it was large between those two membranes and the FA 6-4 P10 membrane, which may have resulted from the difference in fiber collection time during electrospinning. The electrospinning of the FA 6-4 P10 membrane took longer than the other two membranes. In the case of fixing the polymer percentage and increasing the acetone weight fraction, the membrane became thinner. The thickness of the FA 5-5 P10 and FA 4-6 P10 membranes sharply decreased to 103 and 77.5 µm, respectively, compared with the FA 6-4 P10 membrane. In addition, the NIPS membrane was the thinnest one compared to all nanofiber membranes. In the same way, the increase in acetone weight fraction affected the mean pore size by making it narrower. The FA 4-6 P10 membrane with the highest amount of acetone possessed a mean pore size that was smaller than the FA 5-5 P10 and FA 6-4 P10 membranes. The polymer percentage had little effect on the mean pore size, but all membranes, in this case, recorded mean pore sizes in the same range between 202 and 218. However, the mean pore sizes of all the prepared membranes were found to be in a good range—lower than 0.3 µm (300 nm). The results showed that the thickness of the membrane is directly proportional to the viscosity of the dope solution. So, with the increase in the viscosity of dope solutions, the thickness of the membranes increases and vice versa. In addition, especially when the polymer percentage is fixed, a thinner nanofiber membrane tends to form a smaller pore size [43].

**Figure 3:**
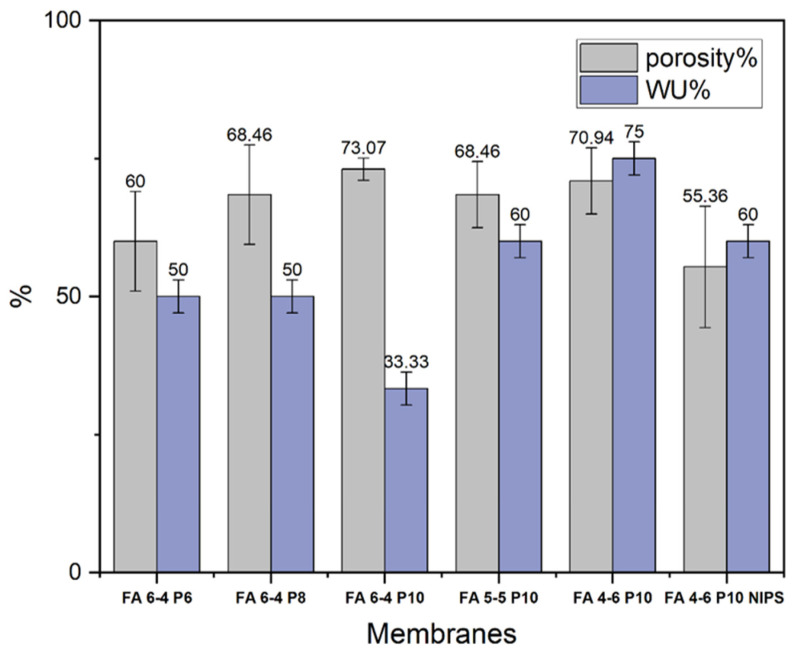
Porosities and WU of PVDF-HFP electrospun membranes.

The porosity of electrospun PVDF-HFP membranes was tested in kerosene and the results are shown in Figure 3. Firstly, the porosity of nanofiber membranes increased as the PVDF-HFP percentage increased, the highest of which was recorded for the FA 6-4 P10 membrane. The porosity of FA 6-4 P10 was 73.07%, while the porosities of the FA 6-4 P8 membrane and FA 6-4 P6 membrane were 68.46.16% and 60%, respectively. In the case of fixed polymer percentage, the porosities of three membranes (FA 6-4 P10, FA 5-5 P10, and FA 4-6 P10) were in the same range and were not largely affected by the increasing acetone concentration. Unlike water uptake, the water uptake of the nanofiber membranes increased with the increase in acetone weight fraction. The lowest percentage of water uptake was 33.3%, which was recorded by FA 6-4 P10. The water uptake increased to 60 and 75% when the acetone weight fraction was increased in the FA 5-5 P10 and FA 4-6 P10 membranes, respectively. Generally, all nanofiber membranes possess excellent porosity greater than 60%.

**Figure 4:**
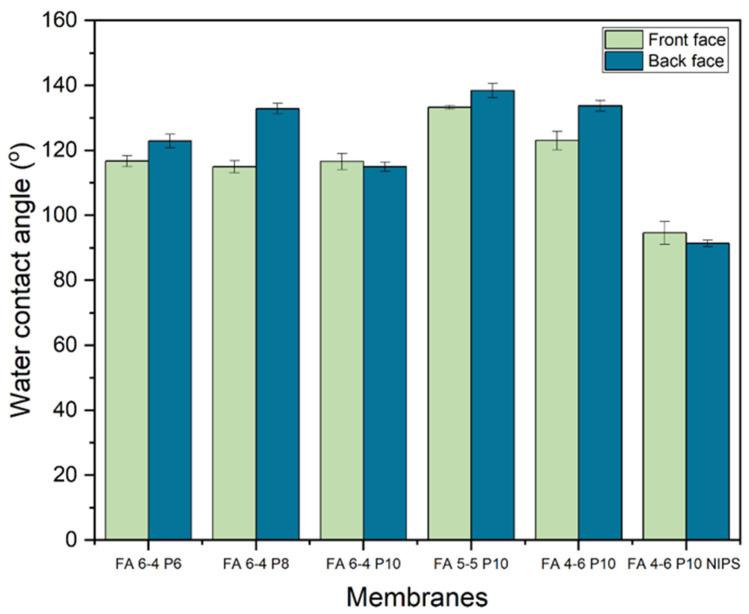
Water contact angles of PVDF-HFP electrospun membranes.

The hydrophobicity of the membranes was also tested by measuring the water contact angle for each face of the membrane. The results showed that the back face of almost all the membranes had a higher hydrophobicity property. As shown in Figure 4, the highest contact angle was recorded by DMF/acetone membranes in two ratios of 5:5 and 4:6 (FA 5-5 P10 and FA 4-6 P10) on both sides, which were (133.22° and 138.39°) and (122.98° and 133.66°) for the front and back faces, respectively. Among membranes containing 10% of PVDF, the lowest contact angle was recorded by the one with a weight ratio of 6:4 which was 116.56° for the front face and 114.95° for the back face. When the solvent weight ratio of DMF/acetone was fixed at 6:4 with a changing PVDF-HFP percentage (FA 6-4 P6, FA 6-4 P8, and FA 6-4 P10), the back face of all membranes showed a superior water contact angle compared with the front face. According to the back face contact angle of DMF/acetone membranes, the best percentage of PVDF-HFP is 8% since its contact angle was 132.82°, whereas in the FA P6 and FA P10 membranes, it was 122.75° and 114.95° for the back face, respectively. On the other hand, the front faces of the FA 6-4 P6 and FA 6-4 P10 membranes had almost the same contact angle of 116.65° and 116.56°, respectively. However, the front face contact angle of the FA 6-4 P8 membrane was significantly lower than the back face. Nonetheless, all membranes were sufficiently hydrophobic as their water contact angles were greater than 90° [44].

**Figure 5:**
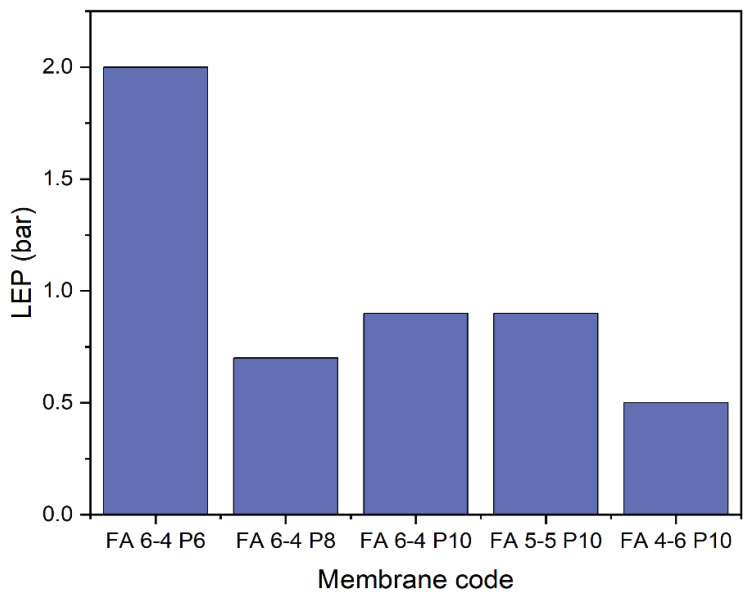
LEP values of PVDF-HFP electrospun membranes.

As shown in the literature, the LEP value of the membrane is directly influenced by the membrane thickness and pore size, and this explains the fluctuation of LEP values. A high LEP value refers to a low pore size and the optimum thickness of the membrane. Figure 5 shows membrane LEP values. The membranes with different polymer weights (FA 6-4 P6, FA 6-4 P8, and FA 6-4 P10) showed that membrane with the lowest polymer percentage (FA 6-4 P6) had the highest LEP, which resulted from its lower pore size. The LEP value increased from 0.7 to 0.9 bar with the polymer percentage increasing from 8% in the FA 6-4 P8 membrane to 10% in the FA 6-4 P10 membrane, as a result of a decrease in pore size and increased thickness. In the case of an equal weight fraction of DMF and acetone, the LEP value remained at 0.9 bar for the FA 6-4 P10 membrane, even though the FA 6-4 P10 membrane had a much higher thickness compared to the FA 5-5 P10 membrane, and this difference may have disappeared because the FA 6-4 P10 membrane possessed a larger pore size than the FA 5-5 P10 membrane, which accelerated the wettability and reduced the LEP value. In contrast, when the weight fraction of acetone was higher than that of DMF (FA 4-6 P10), the LEP value reduced to 0.5 bar, since the thickness of the membrane significantly decreased but the pore size slightly decreased [32].

**Figure 6:**
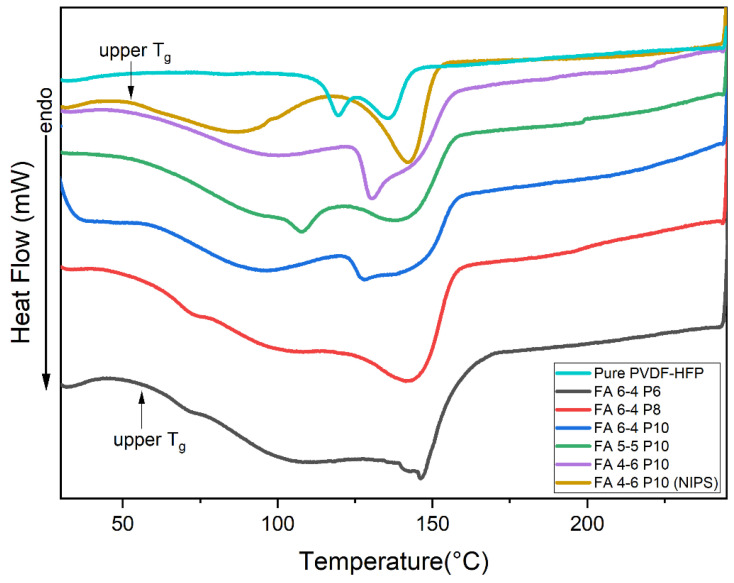
DSC thermograms of PVDF-HFP electrospun nanofiber membranes.

The crystalline phase composition and thermal properties of pure PVDF-HFP and nanofiber membranes were investigated using the DSC technique. The DSC thermograms of PVDF-HFP and the nanofiber membranes are presented in Figure 6. It should be noted that the glass transition temperature (Tg) of PVDF-HFP is around −35 °C, as mentioned in the literature [45]. From the DSC curves, thermodynamic parameters were calculated and listed in Table 6. As shown in Table 6, the increase in PVDF-HFP content led to an increase in melting enthalpy and crystallinity percentage, which was observed in three membranes of FA 6-4 Px (x = 6, 8, and 10). FA 6-4 P6 had a very low melting enthalpy and crystallinity percentage compared to FA 6-4 P8 and FA 6-4 P10. The values of melting enthalpy and crystallinity of membranes containing 8 and 10% were very close. Thus, in membranes produced with a high polymer percentage, the crystalline region was higher than the amorphous region. Overall, the crystallinity of all nanofiber membranes was in the very low range and lower than the crystal content of pure PVDF-HFP.

The pure PVDF-HFP curve, as shown in Figure 6, has two peaks appearing at 119 and 135 °C, which relate to the α-phase and β-phase, respectively. However, the nanofiber membrane curves have only one peak, with the other appearing as a broad low peak or broad drop in the curve line. An endothermic peak appeared between 130 and 148 °C in different nanofiber membranes due to PVDF-HFP copolymer melting. In addition, compared to the curve of pure PVDF-HFP, it is also related to the presence of the β-phase, whereas the disappearance of the first peak (at 119 °C) resulted from the absence of the α-form. The broad drop in the curve line of nanofiber membranes has been considered a second T_g_ (upper T_g_), and it may indicate reorganization inside α-crystals, the melting of paracrystalline domains, or molecular motions corresponding to an α-relaxation in the crystalline/amorphous interface [46]. All these explanations make sense with the disappearance of the α-phase peak. Some studies also reported that the first curve that appeared in the range of 50 to 125 °C in different nanofiber membranes was considered a peak, and it refers to the presence of residual moisture [47,48]. The other suggested that the double peak of melting is attributed to its polymorphic structure; it was also referring to the presence of recrystallization of molten polymer and imperfect crystals in pure PVDF-HFP. Furthermore, it may be related to the difference in the arrangement of bonding such as “head-to-head” or “tail-to-tail” in PVDF-HFP membranes, which affects their thermodynamic behavior and crystalline phase formation [49,50]. Further research revealed that this curve is not considered an individual peak but rather a drop caused by phase transition [51]. Overall, the DSC result concluded that the amorphous phase dominates in all nanofiber membranes and the crystallinity is very low.

**Figure 7:**
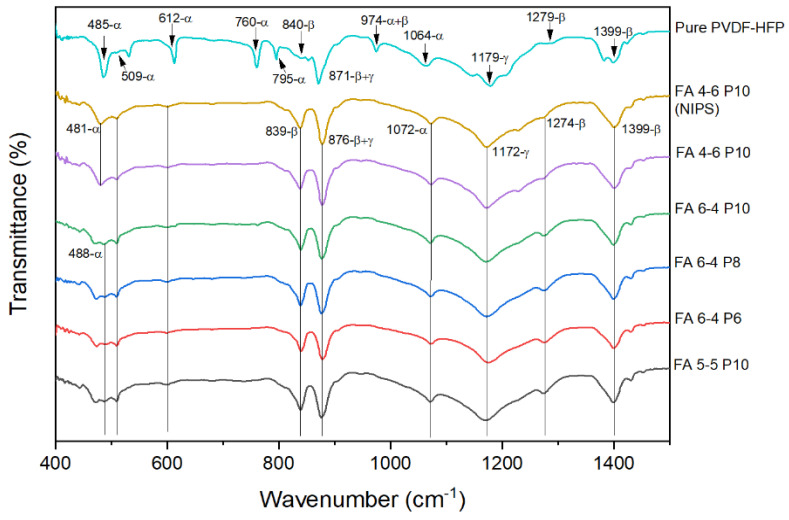
FTIR results of PVDF-HFP electrospun membranes.

The FTIR results of different nanofibers are presented in Figure 7. FTIR peaks give information about the functional groups and crystalline phases that exist in membranes. Thus, the vibrational peak at 471 cm^−1^ is attributed to C–F wagging vibrations. The bands observed at 839 cm^−1^ and 795 cm^−1^ are assigned to CH_2_ rocking vibrations, while its swinging vibration was shown at 1179 cm^−1^. The vibrational peaks at 509 and 1279 cm^−1^ are assigned to the bending and asymmetric stretching vibrations of the CF_2_ group. The peak observed at 1399 cm^−1^ is related to the wagging vibration of CH_2_ or stretching vibrations of the CF group. All these vibrational peaks were found in the FTIR spectra of pure PVDF-HFP and all nanofiber membranes. Besides that, the crystallinity of pure PVDF-HFP and all nanofiber membranes was illustrated by FTIR and XRD peaks. The crystalline content of PVDF is less than 60%, as mentioned in the literature. Nonetheless, the amorphous part is not well studied and no trusted information about how it affects the XRD and FITR spectra can be found. So, this part focused on describing the crystalline phases of pure PVDF-HFP and its nanofiber membranes. The peaks of FTIR and XRD spectra describe the nonpolar crystalline (i.e., α-phase) and polar β- and γ-phases. The nonpolar crystalline α-phase of pure PVDF-HFP was observed at 1064, 795, 760, 612, 509, and 485 cm^−1^, while the peaks at 1300, 1279, and 839 cm^−1^ were assigned to amorphous β-phase and 1179 cm^−1^ for the γ-phase. The dual character peaks that appeared at 975 were due to a mixture of α- and γ-phases, while 871 cm^−1^ was attributed to a mixture of β- and γ-phases [23,52,53,54]. As shown in Figure 7, some peaks of nonpolar crystalline α-phase disappeared in the spectra of membranes which were exhibited in pure PVDF-HFP spectra; but still, the other peaks of α-phase appeared, although with lower intensity (i.e., 485, 509, 612, and 1064 cm^−1^). Furthermore, all the vibrational peaks of the amorphous polar β-phase and semipolar γ-phase appeared more intensely and broadly compared to the same peaks in the pure PVDF-HPF spectra. However, these results reveal that all membranes contain an amorphous polar β-phase and semipolar γ-phase and a very slight amount of nonpolar α-phase. Changing the solvent weight ratio in DMF/acetone binary solvents could influence the volatility of the solvent system, consequently affecting the evaporation rate during electrospinning. The vapor pressure of each solvent system indicates the evaporation rate, which increased as the acetone weight ratio increased. In summary, as the volatility, vapor pressure, evaporation rate, or voltage are increased during electrospinning, so does the formation of the β-phase in the produced membrane [55,56].

**Figure 8:**
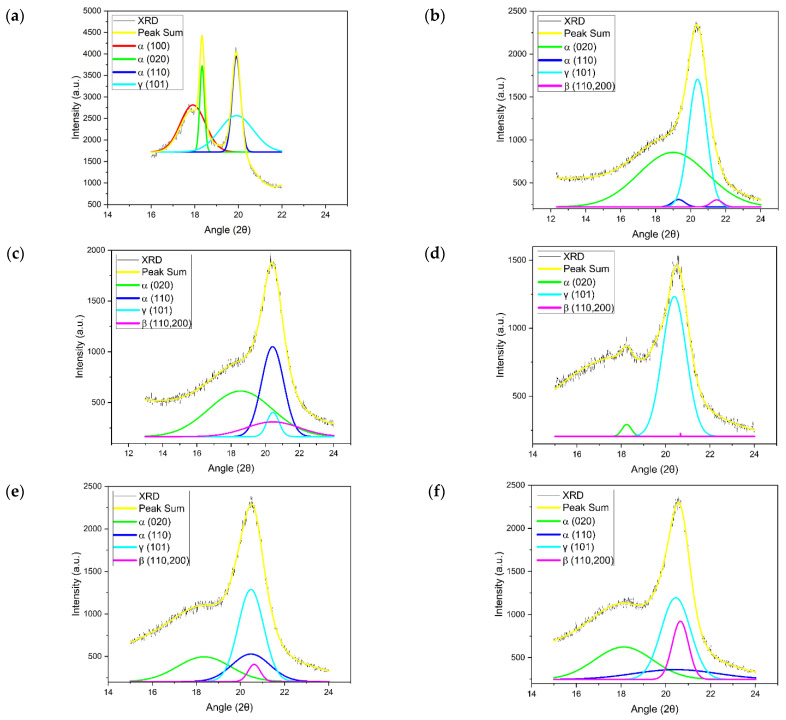
A typical deconvoluted XRD patterns for α-phase (green and dark blue lines), β-phase (purple line), and γ-phase (light blue line) of (**a**) pure PVDF-HFP; (**b**) FA 6-4 P6; (**c**) FA 6-4 P8; (**d**) FA 6-4P10; (**e**) FA 5-5 P10; (**f**) FA 4-6 P10.

The XRD spectra of pure PVDF-HFP and nanofiber membranes provided good agreement with the FTIR results, where XRD spectra revealed peaks corresponding to a crystalline nonpolar α-phase and amorphous polar β- and γ-phases, as FTIR spectra had previously revealed. The XRD spectra of nanofiber membranes are presented in Figure 8. The strong peaks at 17.6, 18.4, and 19.9 correspond to 100, 020, and 110, respectively, which are related to the nonpolar α-phase. This finding demonstrates that the α-phase exists as the primary phase in powder PVDF-HFP. This does not mean that the PVDF-HFP contains only an α-phase, but also contains the γ- and β-phases, which appeared in weak peaks at 20.3 and 20.9, corresponding to 101 and 110/200, respectively. All PVDF-HFP electrospun nanofiber membranes showed peaks of the α-phase and amorphous polar β- and γ-phases [24,57]. Finally, DSC and FTIR confirmed XRD results; all nanofiber membranes exhibited a semicrystalline nature.

### 3.3. Morphological Studies

**Figure 9:**
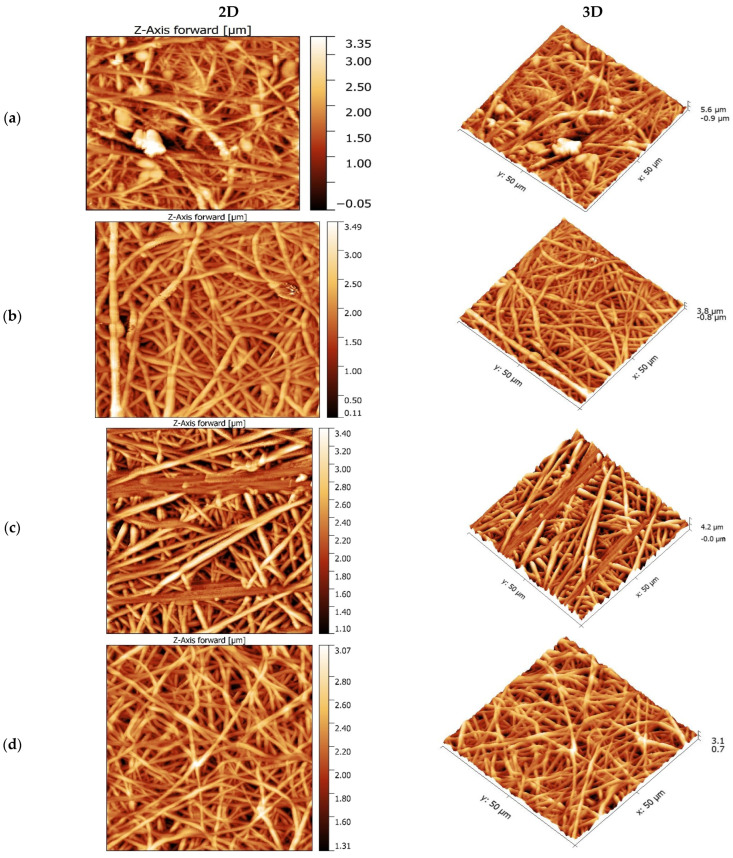

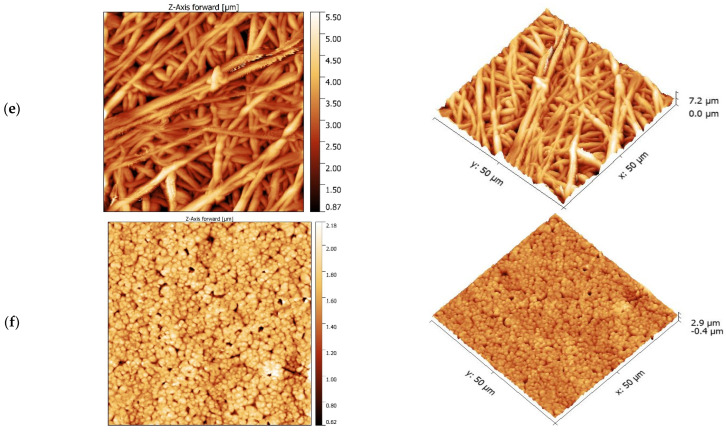
Two-dimensional and three-dimensional AMF images of (**a**) FA 6-4 P6; (**b**) FA 6-4 P8; (**c**) FA 6-4P10; (**d**) FA 5-5 P10; (**e**) FA 4-6 P10; and (**f**) FA 4-6 P10 (NIPS) membranes.

To evaluate the morphological structure of PVDF-HFP nanofiber membranes, the AFM technique was used. It was used to examine the internal construction of polymeric nanofibers and determine the average fiber diameter (AFD). From the 3D AFM images (Figure 9), the average surface roughness (R_a_) and root mean square roughness (R_q_) were calculated for PVDF-HFP nanofiber membranes and are listed in Table 7. AFM found that the average surface roughness of the nanofiber membranes increased with polymer percentage. The higher average surface roughness was recorded by the membrane produced with the highest weight ratio of acetone (FA 4-6 P10), which agreed with its contact angle (133.66°). When the weight ratio of DMF and acetone was equal, the average surface roughness sharply decreased to 258.1 nm. In the same way, the root mean square roughness was 697.8 nm for the FA 6-4 P10 membrane, which was the highest value. Since the FA 6-4 Px (x = 6 and 8) polymeric solution had a high surface tension of 38.5 and 39.52 mNm^−1^, it formed many beads in the fibers. Because the surface tension of the polymeric solution in the FA 6-4 P10 membrane was lower, this effect vanished. Thus, a lower polymer concentration always increases the bead formation and produces nonuniform fibers. This can be concluded by the fact that when the surface tension is lower or equal to 37 mNm^−1^, such as in the case of the FA 6-4 P10, FA 5-5 P10, and FA 4-6 P10 polymeric solutions, smooth and uniform nanofibers will be produced. When the surface tension is above 37 mNm^−1^, beads start to form on nonuniform fibers and nanofiber networks that are heterogeneous, as shown in Figure 9a–c. In addition, polymer concentration is an essential parameter that affects the polymeric solution’s way of spinning in the electrospinning process and, consequently, the morphology of fibers in electrospun membranes.

The FA 6-4 P6 membrane exhibited heterogeneous networks with nonuniform beaded nanofibers, while the nanofibers improved in the 6-4 P8 membrane, where the fiber was more regular and the formation of the beads on the fibers was decreased. However, the nanofiber networks were almost organized (homogeneous), with smoother fibers in the membrane produced with a high polymer percent of FA 6-4 P10 (high viscosity). Polymeric solutions with a high polymer percentage had a good viscoelastic force that made the jet during electrospinning continuously elongate due to it fitting the coulombic and electrostatic forces, whereas the jet of the polymeric solution with a low polymer percentage underwent partial fracture because its viscoelastic force was low and could not fit the other forces that affect the electrospinning operation [29,41]. On the other hand, the beads did not form on the nanofibers of the FA 5-5 P10 and FA 4-6 P10 membranes, as observed in Figure 9d,e, and the nanofibers were perfectly entangled. The viscosities of the polymeric solutions (375 and 339.5 cp for FA 5-5 P10 and FA 4-6 P10) are attributed to this effect because they are higher than the viscosities of AF 6-4 P6 and FA 6-4 P8, which have viscosities of 70 and 175 cp and do not have these features. The second thing that may be responsible for this behavior is the solvent evaporation rate during the electrospinning process which is influenced by the vapor pressure and volatility property of the solvent. DMF has a low vapor pressure and volatility, whereas acetone has a high vapor pressure and volatility; therefore, different solvents’ mixing composition resulted in different volatility and vapor pressure. In addition, the smaller fiber diameter produced by the solution possesses low viscosity and low vapor pressure (high boiling point) [58]. The requirement for low viscosity is to produce fine fibers to prevent polymer macromolecules from becoming entangled, as occurs in solutions with high viscosity [41]. In addition, the morphology of the NIPS membrane in Figure 9f differs from all other membranes (Figure 9a–e). The NIPS membrane shows a spongy texture, while the electrospun membranes show nanofibers. The roughness of the NIPS membrane was sharply decreased to be at the lowest level compared to all electrospun nanofiber membranes, and the lowest difference in Ra values between them was 144 nm. These observations explain the previous contact angle results, which showed that the contact angle of the NIPS membrane was much lower than the contact angle of all electrospun nanofiber membranes.

### 3.4. DCMD Application

MD tests were used to evaluate the performance of different PVDF-HFP nanofiber membranes. The feed side used brine water at 70 °C, and the permeate temperature was 20 °C. Water vapor passed through the pores of the membranes due to the difference in vapor pressure between the two sides. The NIPS membrane did not give any flux of water under these experimental conditions because it had a very small pore size and very low porosity. Table 8 shows the average water flux and salt rejection of different nanofiber membranes. Since the FA 6-4 P6 membrane exhibited a larger pore size and higher porosity than the FA 6-4 P8 and FA 6-4 P10 membranes, it also exhibited good permeability in the MD test. 

**Figure 10:**
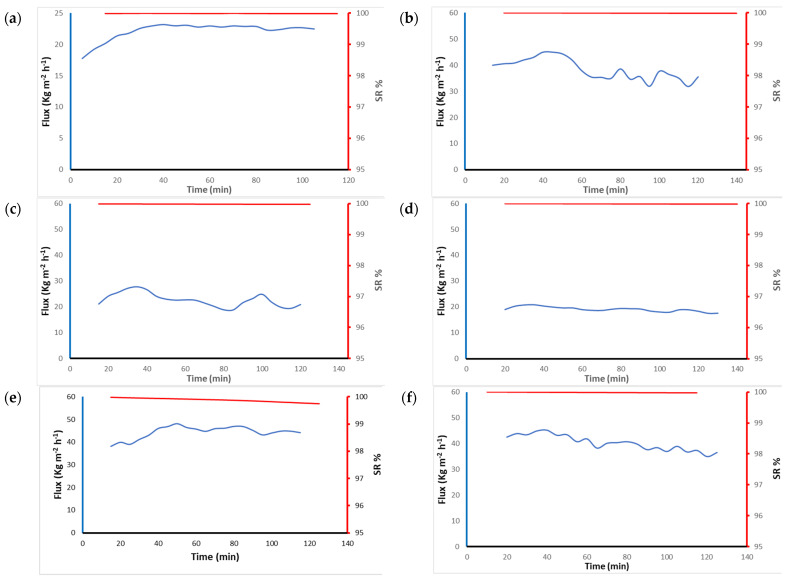
DCMD water flux (blue line) and salt rejection (red line) vs. time plot for (**a**) PVDF; (**b**) FA 6-4 P6; (**c**) FA 6-4 P8; (**d**) FA 6-4P10; (**e**) FA 5-5 P10; and (**f**) FA 4-6 P10.

As can be seen in Figure 10, the flux of water was maintained above 25 kg m^−2^ h^−1^ during the two-hour test by three different membranes: FA 6-4 P6, FA 5-5 P10, and FA 4-6 P10. They achieved a higher water flux compared to the PVDF membrane filter disc. According to the average water flux listed in Table 8, the PVDF membrane filter disc provided 22 kg m^−2^ h^−1^, while FA 6-4 P6, FA 5-5 P10, and FA 4-6 P10 were all greater than 38 kg m^−2^ h^−1^. The other two membranes, FA 6-4 P8 and FA 6-4 P10, gave average water flux values very close to those of the PVDF membrane filter disc and showed almost the same behavior. The behavior of the FA 6-4 Px (x = 6, 8, and 10) membranes in the MD test shows that as the PVDF-HFP content increased, the water flux decreased. This result is expected due to the hydrophobicity property of PVDF-HFP, which increased as the polymer content rose. The roughness of membranes also increased as the polymer content increased, which made the membranes have high hydrophobicity and made it difficult for vaporized water to pass across the membrane pores. Since the porosity is directly proportional to the permeability of the membrane, this provided a good argument for these results. As mentioned previously, the porosities of the FA 6-4 Px (x = 6, 8, and 10) membranes were 81.27, 79.16, and 63.45%, respectively; consequently, the permeability was found to be higher in the FA 6-4 P6 membrane than in the FA 6-4 P8 membrane and at a lower level in the FA 6-4 P10 membrane. The permeability behavior of the FA 6-4 Px (x = 6, 8, and 10) membranes in the first 50 min was constant; after that, it slightly decreased, except for the FA 6-4 P10 membrane. On the other hand, even though the porosity of the FA 6-4 P10 membrane was higher than the porosities of the FA 5-5 P10 and FA 4-6 P10 membranes by 5%, the permeability of the FA 5-5 P10 and FA 4-6 P10 membranes increased by approximately 20 kg m^−2^ h^−1^ compared to the permeability of the FA 6-4 P10 membrane.

The average salt rejection of PVDF-HFP nanofiber membranes was found to be above 90%, as shown in Table 8, which proves the efficiency of these nanofiber membranes in water treatment and contamination removal. The FA 5-5-P10 membrane achieved the perfect performance in terms of permeability and salt rejection. Even though a lower water flux in the MD test was achieved with the FA 6-4 P8 and FA 6-4 P10 membranes, they achieved a higher salt rejection percentage (99.9%). The FA 6-4 P6 and FA 4-6 P10 membranes had a higher water flux but slightly lower salt rejection (91.5% and 91.7%, respectively) compared to the others. Regardless, during the MD experiments, all PVDF-HFP electrospun nanofiber membranes demonstrated good permeability and salt rejection for two hours with no damaged or sharply increased conductivity.

## 4. Conclusions

In this work, PVDF-HFP nanofiber membranes were prepared with different solvent ratios and different polymer percentages through electrospinning. The preliminary tests for binary solvent compositions indicated that these compositions could be used in electrospinning to produce nanofiber membranes. A series of characterization tests were used to examine the ability to utilize these membranes in water treatment and predict their behavior during DCMD. The permeability of membranes was tested in terms of pore size and porosity, from which it can be concluded that all the membranes had narrow pores and good porosity. The LEP values between 2 and 0.5 bar and contact angle results indicated that all the electrospun nanofiber membranes had good hydrophobicity. The DSC thermogram curves of the PVDF-HFP nanofiber membranes showed different behavior than the pure PVDF-HFP. Two thermal peaks appeared in the curve of the pure PVDF-HFP, but in the curves of the nanofiber membranes, one thermal peak appeared with a broad curve, which was difficult to explain. Until now, the crystallinity of the copolymer PVDF-HFP has been unclear, and efforts should be directed towards describing the broad thermal curve in DSC curves, as well as classifying DSC peaks according to phase (polar α-phase and amorphous polar β- and γ-phases).

The DSC, FTIR, and XRD results confirmed that all the nanofiber membranes were semicrystalline with polar α-phases and amorphous polar β- and γ-phases. The NIPS membrane showed poor characteristics compared to the electrospun nanofiber membranes, where its porosity, thickness, and water contact angle were much lower than those of the nanofiber membranes. Because the NIPS membrane had very low porosity, its LEP could not be calculated and could not be applied in the DCMD. In the DCMD, almost all the membranes provided good results, but the highest water flux with constant high salt rejection (99.8%) was observed only with the FA 5-5 P10 membrane. The efficiency of PVDF-HFP nanofiber membranes for water treatment purposes was studied with brine water, but it can be tested with other types of wastewater. 

This study recommends that the membranes discovered to be good are long-term-tested and their performance is improved by enhancing their hydrophobic properties via the insertion of nanoparticles on their surfaces. 

## Figures and Tables

**Table 1:** Description of preparation parameters of each dope solution, which are named according to the weight fraction of solvents and polymer percent.

Dope Solution Abbreviation	Weight Ratio (wt%) of Solvents		PVDF-HFP %	LiCl %
	DMF	Acetone		
FA 6-4 P6	6	4	6	0.1
FA 6-4 P8	6	4	8	0.1
FA 6-4 P10	6	4	10	0.1
FA 5-5 P10	5	5	10	0.1
FA 4-6 P10	4	6	10	0.1

**Table 2:** Properties and Hansen solubility parameters for solvents and PVDF-HFP [17].

Solvents and Polymer	Vapor Pressure (kPa)	Surface Tension (mN m^−1^)	Hansen Solubility Parameters			
			*δ_d_* (MPa^1/2^)	*δ_p_* (MPa^1/2^)	*δ_h_* (MPa^1/2^)	*R*_0_ (MPa^1/2^)
**Acetone**	25	23	7.6	5.1	3.4	-
**DMF**	0.38	37	17.4	13.7	11.3	-
**DMSO**	0.056	43.5	18.4	16.4	10.2	-
**PVDF**	-	-	17.2	12.5	8.2	9.6

**Table 3:** Properties of solvent systems.

Solvent Systems	FA 6-4	FA 5-5	FA 4-6
**Solubility parameter (MPa)^1/2^**	4.34	5.69	7.11
***RED***	0.45	0.59	0.74
**Vapor pressure (kPa)**	11.59	14.08	16.46

**Table 4:** Viscosity and surface tension properties of PVDF-HFP dope solutions.

Dope Solutions	FA 6-4 P6	FA 6-4 P8	FA 6-4 P10	FA 5-5 P10	FA 4-6 P10
**Viscosity (cp)**	70	175	405	375	339.5
**Surface tension** **(mN m^−1^)**	38.5	39.52	37.82	37.24	35

**Table 5:** Mean thickness and pore size results of PVDF-HFP electrospun nanofiber membrane.

Membrane	Mean Thickness (µm)	Mean Pore Size (nm)
**FA 6-4 P6**	64.8 ± 7.8	218 ± 5
**FA 6-4 P8**	184 ± 11.4	203 ± 5
**FA 6-4 P10**	251 ± 15.7	217 ± 5
**FA 5-5 P10**	103 ± 18.9	180 ± 5
**FA 4-6 P10**	77.5 ± 18.7	178 ± 5
**FA 4-6 P10 NIPS**	50.75 ± 4.2	-

**Table 6:** Thermal properties from DSC analysis of pure PVDF-HFP and nanofiber membranes that were prepared from either different polymer percents or different weight ratios of solvents.

Membranes	Pure PVDF-HFP	FA 6-4 P6	FA 6-4 P8	FA 6-4 P10	FA 5-5 P10	FA 4-6 P10	FA 4-6 P10 (NIPS)
**T_m_ (°C)**	119.90 135.37	148.0	144.0	142.5	142.1	130.6	142.32
**Upper T_g_ (°C)**	-	67.36	66.98	72.07	73.5	79.23	56.61
**ΔH_m_ (J g^−1^)**	12.10	3.954	9.705	8.62	12.35	11.48	10.76
**X%**	11.56	3.77	9.26	8.23	11.79	10.96	10.27

**Table 7:** Roughness parameters for various PVDF-HFP nanofiber membranes.

Membranes	R_a_ (nm)	R_q_ (nm)	AFD (µm)
**FA 6-4 P6**	284.1	367.83	1.20 ± 0.5
**FA 6-4 P8**	373.84	477.06	1.21 ± 0.2
**FA 6-4 P10**	382.06	455	1.78 ± 0.4
**FA 5-5 P10**	258.01	324.33	0.93 ± 0.5
**FA 4-6 P10**	593.83	697.8	1.67 ± 0.4
**FA 4-6 P10 (NIPS)**	114.39	136.46	-

**Table 8:** Average water flux and salt rejection of PVDF-HFP electrospun nanofiber membranes during the MD test.

Membrane Code	Average Water Flux (kg m^−2^ h^−1^)	Average Salt Rejection (%)
**Commercial PVDF membrane**	22.1 ± 1.4	99.9
**FA 6-4 P6**	38.3 ± 4.0	91.5
**FA 6-4 P8**	22.7 ± 2.6	99.9
**FA 6-4 P10**	19.1 ± 0.9	99.9
**FA 5-5 P10**	44.4 ± 2.9	99.8
**FA 4-6 P10**	40.3 ± 2.9	91.7

## Data Availability

Not applicable.

## Abbreviations

MD	Membrane distillation
RO	Reverse osmosis
MF	Microfiltration
UF	Ultrafiltration
NF	Nanofiltration
FO	Forward osmosis
PVDF-HFP	Polyvinylidene fluoride-co-hexafluoropropylene
PVDF	Polyvinylidene fluoride
DCMD	Direct-contact membrane distillation
VMD	Vacuum membrane distillation
AGMD	Air gap membrane distillation
SGMD	Sweeping gas membrane distillation
LEP	Liquid entry pressure
T_g_	Glass transition temperature
DMSO	Dimethyl sulfoxide
DMF	Dimethylformamide
LiCl	Lithium chloride
NIPS	Nonsolvent-induced phase separation method
CA	Contact angle
WU	Water uptake
DSC	Differential scanning calorimetry
XRD	X-ray diffraction
FT-IR	Fourier-transform infrared
RED	Relative energy density
AFM	Atomic force microscopy
WU	Water uptake
AFD	Average fiber diameter
SWRO	Seawater reverse osmosis
